# Early initiation of insulin in a child with T2DM and clinical course - a case report

**DOI:** 10.1186/1687-9856-2013-S1-P26

**Published:** 2013-10-03

**Authors:** S Ramkumar, AC Ammini

**Affiliations:** 1 Dept of Endocrinology and Metabolism, AIIMS, New Delhi, India

## Aim

To report a 13 year old girl with T2DM who had been initiated on insulin and the course of her glycemic status.

## Methods

We describe a case of T2DM in a 13 year old girl – her clinical presentation, diagnosis and change in glycemic control over a period of 12 months.

## Results

13 year old presented with osmotic symptoms for 3 weeks duration. There was no change in weight. She had a strong family history of DM in mother and grandmother. On examination, general examination was unremarkable with no acanthosis nigricans, skin tags, or visceral obesity. Her vitals were normal. Her BMI was 23.42 kg/m^2^. Her initial blood glucose levels are fasting 245 mg% and post prandial 340mg%. Her urine ketones were negative and her initial HbA1c was 8.3%. Her fasting insulin and c-peptide levels (before initiation of insulin treatment) were 101µU/ml (2.6 – 24.9) and 8.58 ng/ml (1.1 – 4.4) respectively. Her TPO and GAD antibody were negative. Her hemogram, liver, renal and thyroid functions were normal.

She was initiated treatment with inj.premix (30/70) 12 units before breakfast (BBF) and 10 units before dinner (BD). Over the course of next 1 year, her insulin requirement was gradually tapered to 6 units BBF and 6 units BD. Her current glycemic status was fasting 93 mg% and post prandial 76mg% with HbA1c of 6.5%. Insulin and c-peptide levels were 12.6 µU/ml (2.6 – 24.9) and 1.8 ng/ml (1.1 – 4.4). She had gained 5cm height and lost 2 kg in last 1 year.

## Results

As per the ADA( American Diabetes Association) and ISPAD (International Society of Pediatric and Adolescent Diabetes) recommendations, insulin is currently not recommended as initial treatment in the management of children with T2DM unless they present with ketosis or Hba1C >8.5%. However, this case scenario shows insulin when initiated early in children with newly diagnosed diabetes in a state of glucotoxicity, when response to oral hypoglycemic may be suboptimal, may have better glycemic control even at lower doses.

